# Confidence intervals for the common coefficient of variation of rainfall in Thailand

**DOI:** 10.7717/peerj.10004

**Published:** 2020-09-21

**Authors:** Warisa Thangjai, Sa-Aat Niwitpong, Suparat Niwitpong

**Affiliations:** 1 Department of Statistics, Faculty of Science, Ramkhamhaeng University, Bangkok, Thailand; 2 Department of Applied Statistics, Faculty of Applied Science, King Mongkut’s University of Technology North Bangok, Bangkok, Thailand

**Keywords:** Coefficient of variation, Lognormal distribution, Common coefficient of variation, Dispersion of rainfall, Climate sciences and hydrology

## Abstract

The log-normal distribution is often used to analyze environmental data like daily rainfall amounts. The rainfall is of interest in Thailand because high variable climates can lead to periodic water stress and scarcity. The mean, standard deviation or coefficient of variation of the rainfall in the area is usually estimated. The climate moisture index is the ratio of plant water demand to precipitation. The climate moisture index should use the coefficient of variation instead of the standard deviation for comparison between areas with widely different means. The larger coefficient of variation indicates greater dispersion, whereas the lower coefficient of variation indicates the lower risk. The common coefficient of variation, is the weighted coefficients of variation based on *k* areas, presents the average daily rainfall. Therefore, the common coefficient of variation is used to describe overall water problems of *k* areas. In this paper, we propose four novel approaches for the confidence interval estimation of the common coefficient of variation of log-normal distributions based on the fiducial generalized confidence interval (FGCI), method of variance estimates recovery (MOVER), computational, and Bayesian approaches. A Monte Carlo simulation was used to evaluate the coverage probabilities and average lengths of the confidence intervals. In terms of coverage probability, the results show that the FGCI approach provided the best confidence interval estimates for most cases except for when the sample case was equal to six populations (*k* = 6) and the sample sizes were small (*n*_*I*_ < 50), for which the MOVER confidence interval estimates were the best. The efficacies of the proposed approaches are illustrated with example using real-life daily rainfall datasets from regions of Thailand.

## Introduction

Droughts and floods are regular natural disasters in Thailand. Droughts occur when the hot season begins after a year with unusually little rainfall. Moreover, floods happen nearly every year during the monsoon season. The monsoon seasons in the country are distinct by region. Thailand is divided into six geographical regions such as the north, the northeast, the west, the central, the east, and the south. Various regions are prone to seasonal flash-flooding. The floods often occur in the north, the northern east, and the south. Since rainfall varies greatly depending on region and season. Therefore, the common coefficient of variation is used to represent the rainfall dispersion in different regions.

The log-normal distribution, widely used to describe the distribution of right-skewed data, has been used to model various real-life applications (Jafari & Abdollahnezhad, 2015). For instance, in climate sciences and hydrology, the rainfall measurements are right-skewed (Thangjai, Niwitpong & Niwitpong, 2020a). The coefficient of variation of the log-normal distribution depends on the variance (*σ*^2^) only (Thangjai, Niwitpong & Niwitpong, 2016) whereas the coefficient of variation of normal distribution depends on the mean (µ) and *σ*^2^. Although the normal distribution is more well-known than the log-normal distribution in the natural and social sciences, the latter has been used in many applications. Examples of quantities which have approximate log-normal distributions include the particulate matter and rainfall frequency. Rumburg, Alldredge & Claiborn (2001) studied the statistical distributions of daily particulate matter data from Spokane, Washington from January 1995 to December 1997. They found that the PM2.5 data is best fit by a three parameter log-normal distribution. Thangjai, Niwitpong & Niwitpong (2020a) constructed the simultaneous confidence intervals for all differences of coefficients of variation of daily rainfall data on 17 July 2018 in different regions of Thailand. The daily rainfall data is log-normal distribution.

In statistics, the information in a sample *X* = (*X*_1_, *X*_2_, …, *X*_*n*_) is used to make inferences about an unknown parameter *θ*. The inference methods are hypothesis testing, point estimation, and confidence interval estimation. The statistical hypothesis makes a statement about a population parameter. The hypothesis testing uses the sample from the population for deciding. Two complementary hypotheses in hypothesis testing are the null hypothesis and the alternative hypothesis. The point estimation uses the sample data to evaluate a single value. The point estimation is a guess of the single value as the value of the parameter. The value is called the point estimator. The point estimate is not absolutely accurate because the estimate is based on only the single random sample. For a contrasting point estimation method, the confidence interval estimation uses the sample data to calculate an interval of probable values. The confidence interval is called the confidence interval estimator. The confidence interval estimation is used rather than the point estimation because the confidence interval estimation has some guarantee of capturing the parameter. The goal of this paper is to examine the confidence interval for parameter of log-normal distribution. Confidence intervals associated with various functions of the log-normal distribution parameters have been reported by Land (1988), Zhou & Gao (1997), Zhou (1998), Joulious & Debarnot (2000), Taylor, Kupper & Muller (2002), Krishnamoorthy & Mathew (2003), Gupta & Li (2005), Hannig et al. (2006b), and Shen, Brown & Hui (2006). In these studies, confidence intervals were considered on linear functions of the mean and variance of the log-normal distribution. These results were later extended to stochastic processes such as homogeneous log-normal (Gutiérrez et al., 2007) and non-homogeneous log-normal (Gutiérrez et al., 2003).

The coefficient of variation is defined as the standard deviation divided by the mean (Kelley, 2007). It can be used to compare several populations that have different measurement units and is widely used to measure the precision and repeatability of data in many fields. In hematology and serology, the coefficient of variation has been used for the measurement of blood samples taken from different laboratories (Tsim et al., 1991) and as a measure of precision within and between laboratories (Tian, 2005). In finance, a test of the equality of the coefficients of variation for two stocks has been used to measure risk. In medicine, the coefficient of variation has been used to compare the variability in the ratio of total/high density lipoprotein cholesterol with the variability in vessel diameter change according to diet.

In climate sciences and hydrology, the coefficient of variation has been used to describe the rainfall and can be used to compare the rainfall variability in two or more different areas (Thangjai, Niwitpong & Niwitpong, 2020a). If the difference between the rainfall in a single area and the average rainfall over several areas is high, then the rainfall is high. In statistical analysis, combine the results of several independent studies is used in climate sciences and clinical trial. If it is assumed that the samples are collected from independent log-normal populations with a common coefficient of variation but possibly with different variances, then the confidence interval for the common coefficient of variation of several log-normal populations becomes the parameter of interest. Several researchers have focused on confidence interval estimation for the coefficient of variation of a log-normal distribution. For example, Niwitpong (2013) presented confidence intervals for the coefficient of variation of a log-normal distribution with a restricted parameter space. Ng (2014) proposed an approach to make inference on the common coefficient of variation of log-normal populations. Simultaneous confidence intervals for the differences in the coefficients of variation of log-normal distributions were proposed by Thangjai, Niwitpong & Niwitpong (2016) and Thangjai, Niwitpong & Niwitpong (2020a). Moreover, Nam & Kwon (2017) and Hasan & Krishnamoorthy (2017) studied the confidence intervals for the ratio of coefficients of variation of two log-normal distributions.

Common problems in applied statistics are confidence interval estimation for the coefficient of variation and testing the equality of two or more coefficients of variation. Miller & Karson (1977) proposed a test for the equality of coefficients of variation in two normal populations. Gupta & Ma (1996) presented testing the equality of the coefficients of variation in *k* normal populations. Fung & Tsang (1998) compared parametric and nonparametric tests and Gokpinar & Gokpinar (2015) proposed a computational approach to test the equality of the coefficients of variation of *k* normal populations. Sangnawakij, Niwitpong & Niwitpong (2016) proposed two new confidence intervals for the ratio of the coefficients of variation of two-parameter exponential distributions. Sangnawakij & Niwitpong (2016) presented confidence interval estimation for the single coefficient of variation and the difference between two coefficients of variation in two-parameter exponential distributions.

Under many circumstances, confidence interval estimation or hypothesis testing for the common coefficient of variation based on several independent samples is of interest (Tian, 2005). Krishnamoorthy & Lu (2003) investigated procedures for confidence interval estimation and hypothesis testing of the common mean of several normal populations, while the problem of making inference from common populations with a common coefficient of variation of normal distributions was dealt with by Tian (2005). Tian & Wu (2007) proposed confidence interval estimation and hypothesis testing of the common mean of several log-normal populations using the generalized variable concept. Similarly, procedures for hypothesis testing and confidence interval estimation for the common mean of several inverse Gaussian populations were presented by Ye, Ma & Wang (2010). Moreover, Ng (2014) constructed a confidence interval for the common coefficient of variation from several independent log-normal samples based on the GCI approach, although there was no comparison with other approaches.

The concepts of the generalized pivotal quantity (GPQ) and the GCI first proposed by Weerahandi (1993) have been applied to solve many statistical problems. For example, Krishnamoorthy & Lu (2003) presented the generalized variable and GCI approaches for inference on the common mean of several normal populations. Tian (2005) studied inferences on the common coefficient of variation of several normal populations. Moreover, Tian & Wu (2007) proposed the generalized variable approach and the GCI approach for inferences on the common mean of several log-normal populations. Thangjai, Niwitpong & Niwitpong (2020b) developed confidence intervals for the common coefficient of variation of several normal populations using the GCI and adjusted GCI approaches. Hannig, Iyer & Patterson (2006a) suggested fiducial GPQ (FGPQ) as a subclass of GPQ, with the FGCI being constructed using the FGPQ under fairly general conditions. Furthermore, FGCIs have been constructed to solve many practical problems (Hannig et al., 2006b; Chang & Huang, 2009; Kharrati-Kopaei, Malekzadeh & Sadooghi-Alvandi, 2013; Thangjai, Niwitpong & Niwitpong, 2016). Although the FGCI approach is based on simulated data, one advantage is that it can be used to construct the confidence interval for complex parameters.

The method of variance estimates recovery (MOVER) approach was introduced by Zou & Donner (2008) and Zou, Taleban & Hao (2009). Several researchers have successfully used the MOVER approach to construct confidence intervals (Donner & Zou, 2012; Suwan & Niwitpong, 2013; Niwitpong & Wongkhao, 2016). The MOVER approach has the advantage of being easy to compute using the exact formula. However, the disadvantage of this approach is that one can construct it with or without the initial confidence interval for a single parameter of interest.

The computational approach proposed by Pal, Lim & Ling (2007) has been used by many researchers to test the equality of several populations (e.g., Jafari & Abdollahnezhad, 2015; Jafari & Kazemi, 2017; Gokpinar & Gokpinar, 2017). As an advantage, this approach does not require explicit knowledge of the sampling distribution of the test statistic. However, it is based on simulation and numerical computations using the maximum likelihood estimate only. The Bayesian approach uses Bayes’ theorem to compute and update probabilities after obtaining new data. Although it can be applied to estimate the confidence intervals for complex parameters, the disadvantages of applying it are that it requires prior information and is based on simulation.

Ng (2014) constructed the GCI of a log-normal distribution which used asymptotic variance and provided coverage probabilities close to the nominal confidence level of 0.95. The confidence interval constructed by transforming the log-normal coefficient of variation to the normal coefficient of variation. However, its use was only considered for homogeneous populations. Therefore, in this study, we extend the research of Ng (2014) to develop four novel approaches for confidence interval estimation for the common coefficient of variation of several log-normal populations based on the FGCI, MOVER, computational approach, and Bayesian approaches. Unlike Ng (2014), we compute the confidence intervals for the common coefficient of variation directly using the log-normal coefficient of variation that depends on *σ*^2^ only. Moreover, there is no previous literature on applying their methodology to PM2.5 concentration measurements. Therefore, to fill the gap, the novel approaches for the confidence interval estimation of the common coefficient of variation of log-normal distributions were proposed with considering the log-normality of PM2.5 concentration measurements.

## Methods

Let us consider *Y* the log-normal distribution with parameters *μ*_*Y*_ and ${\sigma }_{Y}^{2}$. It is well known that *X* = log(*Y*) follows a normal distribution with mean µand variance *σ*^2^, whereas the mean and variance of *Y* are given by

(1) \begin{eqnarray*}{\mu }_{Y}=\exp \nolimits \left( \mu + \left( {\sigma }^{2}/2 \right) \right) \end{eqnarray*}

and

(2) \begin{eqnarray*}{\sigma }_{Y}^{2}= \left( \exp \nolimits \left( {\sigma }^{2} \right) -1 \right) \cdot \left( \exp \nolimits \left( 2\mu +{\sigma }^{2} \right) \right) ,\end{eqnarray*}

respectively. From Eqs. (1) and (2), the coefficient of variation of *Y* is given by

(3) \begin{eqnarray*}\theta =\sqrt{\exp \nolimits \left( {\sigma }^{2} \right) -1}.\end{eqnarray*}

From Eq. (3), it is seen that the coefficient of variation of the log-normal distribution depends on parameter *σ*^2^ only, whereas the coefficient of variation of the normal distribution depends on µ and *σ*^2^. And the next result provides a useful approximation for the variance of an estimator of *θ*.

Let $\hat {\theta }=\sqrt{\exp \left( {S}^{2} \right) -1}$ be an estimator of *θ*. Following the Niwitpong (2013) and Hasan & Krishnamoorthy (2017), the variance of $\hat {\theta }$ is

(4) \begin{eqnarray*}Var \left( \hat {\theta } \right) \approx \frac{{\sigma }^{4}\exp \nolimits \left( 2{\sigma }^{2} \right) }{2 \left( n-1 \right) \cdot \left( \exp \nolimits \left( {\sigma }^{2} \right) -1 \right) } .\end{eqnarray*}

Suppose that random samples are taken from *k* log-normal distributions, ${Y}_{i}=({Y}_{i1},{Y}_{i2},\ldots ,{Y}_{i{n}_{i}})\sim LN({\mu }_{i},{\sigma }_{i}^{2})$, where *i* = 1, 2, …, *k*. Let ${\theta }_{i}=\sqrt{\exp \left( {\sigma }_{i}^{2} \right) -1}$ be the coefficient of variation for *i* = 1, 2, …, *k*. Let *X*_*i*_ = (*X*_*i*1_, *X*_*i*2_, …, *X*_*in*_*i*__) be a random variable of size *n*_*i*_ from the normal distribution with *μ*_*i*_ and variance ${\sigma }_{i}^{2}$. Let ${\hat {\mu }}_{i}={\bar {X}}_{i}$ and ${\hat {\sigma }}_{i}^{2}={S}_{i}^{2}$ be estimators of *μ*_*i*_ and ${\sigma }_{i}^{2}$, respectively, where ${\bar {X}}_{i}$ and ${S}_{i}^{2}$ denote the mean and variance of the log-transformed sample from a log-normal distribution. The mean and variance are given by

(5) \begin{eqnarray*}{\bar {X}}_{i}=\sum _{j=1}^{{n}_{i}} \frac{{X}_{ij}}{{n}_{i}} \end{eqnarray*}

and

(6) \begin{eqnarray*}{S}_{i}^{2}= \frac{\sum _{j=1}^{{n}_{i}}{ \left( {X}_{ij}-{\bar {X}}_{i} \right) }^{2}}{{n}_{i}-1} ,\end{eqnarray*}

where *i* = 1, 2, …, *k* and *j* = 1, 2, …, *n*_*i*_.

Let ${\bar {x}}_{i}$ and ${s}_{i}^{2}$ be the observed values of ${\bar {X}}_{i}$ and ${S}_{i}^{2}$, respectively. The maximum likelihood estimator of the coefficient of variation *θ*_*i*_, is also unbiased estimator, is given by

(7) \begin{eqnarray*}{\hat {\theta }}_{i}=\sqrt{\exp \nolimits \left( {S}_{i}^{2} \right) -1},\end{eqnarray*}

where ${S}_{i}^{2}$ is defined in Eq. (6).

### Fiducial generalized confidence interval

The FGCI uses the FGPQs. The FGPQs are a subclass of the GPQs. The FGCI has correct frequentist coverage probability. For *i* = 1, 2, …, *k*, let ${\bar {X}}_{i}$ and ${S}_{i}^{2}$ be the sample mean and the sample variance for log-transformed data and let ${\bar {x}}_{i}$ and ${s}_{i}^{2}$ be the observed sample mean and the observed sample variance respectively. Let ${\bar {X}}_{i}^{\ast }$ and ${S}_{i}^{2\ast }$ be independent copies of ${\bar {X}}_{i}$ and ${S}_{i}^{2}$, respectively. Let ${\bar {X}}_{i}$ and ${\bar {X}}_{i}^{\ast }$ be independent and identically distributed with mean *μ*_*i*_ and variance ${\sigma }_{i}^{2}/{n}_{i}$. It is well known that

(8) \begin{eqnarray*}{\bar {X}}_{i}\sim N \left( {\mu }_{i}, \frac{{\sigma }_{i}^{2}}{{n}_{i}} \right) \text{and} {\bar {X}}_{i}^{\ast }\sim N \left( {\mu }_{i}, \frac{{\sigma }_{i}^{2}}{{n}_{i}} \right) .\end{eqnarray*}

Furthermore, let ${S}_{i}^{2}$ and ${S}_{i}^{2\ast }$ be independent and identically distributed. Then

(9) \begin{eqnarray*} \frac{ \left( {n}_{i}-1 \right) {S}_{i}^{2}}{{\sigma }_{i}^{2}} \sim {\chi }_{{n}_{i}-1}^{2} \text{and} \frac{ \left( {n}_{i}-1 \right) {S}_{i}^{2\ast }}{{\sigma }_{i}^{2}} \sim {\chi }_{{n}_{i}-1}^{2},\end{eqnarray*}

where ${\chi }_{{n}_{i}-1}^{2}$ is chi-squared distribution with *n*_*i*_ − 1 degree of freedom.

According to Hannig, Iyer & Patterson (2006a) and Hannig et al. (2006b), the FGPQs of *μ*_*i*_ and ${\sigma }_{i}^{2}$ are defined by

(10) \begin{eqnarray*}{R}_{{\mu }_{i}}={\bar {X}}_{i}- \frac{{S}_{i}}{{S}_{i}^{\ast }} \left( {\bar {X}}_{i}^{\ast }-{\mu }_{i} \right) \end{eqnarray*}

and

(11) \begin{eqnarray*}{R}_{{\sigma }_{i}^{2}}= \frac{{S}_{i}^{2}}{{S}_{i}^{2\ast }} {\sigma }_{i}^{2}.\end{eqnarray*}

Therefore, the FGPQ for *θ*_*i*_ based on the FGPQ for ${\sigma }_{i}^{2}$ is given by

(12) \begin{eqnarray*}{R}_{{\theta }_{i}}=\sqrt{\exp \nolimits \left( {R}_{{\sigma }_{i}^{2}} \right) -1}.\end{eqnarray*}

The FGPQ of *θ*_*i*_ in Eq. (12) satisfies two conditions defined in Definition of Hannig, Iyer & Patterson (2006a) and Hannig et al. (2006b). The definition of Hannig, Iyer & Patterson (2006a) and Hannig et al. (2006b) has two conditions such as the distribution of the GPQ is free of all unknown parameters and the observed value of the GPQ is the parameter of interest. From Eq. (4), the variance of ${\hat {\theta }}_{i}$ is provided by

(13) \begin{eqnarray*}Var \left( {\hat {\theta }}_{i} \right) \approx \frac{{\sigma }_{i}^{4}\exp \nolimits \left( 2{\sigma }_{i}^{2} \right) }{2 \left( {n}_{i}-1 \right) \cdot \left( \exp \nolimits \left( {\sigma }_{i}^{2} \right) -1 \right) } .\end{eqnarray*}

The FGPQ of $Var \left( {\hat {\theta }}_{i} \right) $ is given by

(14) \begin{eqnarray*}{R}_{Var \left( {\hat {\theta }}_{i} \right) }= \frac{{R}_{{\sigma }_{i}^{2}}^{2}\exp \nolimits \left( 2{R}_{{\sigma }_{i}^{2}} \right) }{2 \left( {n}_{i}-1 \right) \cdot \left( \exp \nolimits \left( {R}_{{\sigma }_{i}^{2}} \right) -1 \right) } .\end{eqnarray*}

The FGPQ for the common coefficient of variation *θ* is a weighted average of the FGPQ *R*_*θ*_*i*__ based on *k* individual sample. Therefore, the FGPQ is given by

(15) \begin{eqnarray*}{R}_{\theta }=\sum _{i=1}^{k} \frac{{R}_{{\theta }_{i}}}{{R}_{Var({\hat {\theta }}_{i})}} \left/ \right. \sum _{i=1}^{k} \frac{1}{{R}_{Var({\hat {\theta }}_{i})}} ,\end{eqnarray*}

where *R*_*θ*_*i*__ is defined in Eq. (12) and ${R}_{Var({\hat {\theta }}_{i})}$ is defined in Eq. (14).

The FGPQ in Eq. (12) satisfies two conditions of the definition given above. The FGCI is constructed using the quantiles of FGPQ defined in Eq. (15). Therefore, the $100 \left( 1-\alpha \right) \text{%}$ two-sided confidence interval for the common coefficient of variation *θ* based on the FGCI approach is

(16) \begin{eqnarray*}C{I}_{FGCI}=[{L}_{FGCI},{U}_{FGCI}]=[{R}_{\theta } \left( \alpha /2 \right) ,{R}_{\theta } \left( 1-\alpha /2 \right) ],\end{eqnarray*}

where ${R}_{\theta } \left( \alpha /2 \right) $ and ${R}_{\theta } \left( 1-\alpha /2 \right) $ denote the $100 \left( \alpha /2 \right) $-th and $100 \left( 1-\alpha /2 \right) $-th percentiles of *R*_*θ*_, respectively.

The following algorithm is used to construct the FGCI:


 
 
 Algorithm 1 
For a given ¯ xi  and s2i, where i = 1,2,...,k 
For g =  1 to m, where m is number of generalized computation 
Generate X∗ and then compute ¯ x∗i  and s2∗i 
Generate χ2ni−1  from chi-squared distribution with ni − 1  degrees of 
freedom 
Compute Rσ2 
i  from Eq.  (11) 
Compute Rθi  from Eq.  (12) 
Compute RV ar(ˆθ 
i)  from Eq.  (14) 
Compute Rθ  from Eq.  (15) 
End g loop 
Compute Rθ (α∕2)  and Rθ (1 − α∕2)  from Eq.  (16)    


### Method of variance estimates recovery confidence interval

Zou & Donner (2008) and Zou, Taleban & Hao (2009) proposed the MOVER approach to construct the confidence interval for the sum of two parameters. For *i* = 1, 2, let *θ*_1_ and *θ*_2_ be the parameters of interest. Let *L* and *U* be the lower limit and upper limit of the confidence interval for *θ*_1_ + *θ*_2_. Moreover, let ${\hat {\theta }}_{1}$ and ${\hat {\theta }}_{2}$ be the estimators of *θ*_1_ and *θ*_2_, respectively. The central limit theorem and the assumption of independence between the point estimates ${\hat {\theta }}_{1}$ and ${\hat {\theta }}_{2}$ are used. Therefore, the lower limit *L* is

(17) \begin{eqnarray*}L={\hat {\theta }}_{1}+{\hat {\theta }}_{2}-{z}_{\alpha /2}\sqrt{\widehat{Var} \left( {\hat {\theta }}_{1} \right) +\widehat{Var} \left( {\hat {\theta }}_{2} \right) },\end{eqnarray*}

where *z*_*α*∕2_ is the $100 \left( \alpha /2 \right) $-th percentile of the standard normal distribution.

Let *l*_*i*_ and *u*_*i*_ be the lower limit and upper limit of the confidence interval for *θ*_*i*_, where *i* = 1, 2. The lower limit *L* must be closer to *l*_1_ + *l*_2_ than to ${\hat {\theta }}_{1}+{\hat {\theta }}_{2}$. The variance estimate for ${\hat {\theta }}_{i}$ at *θ*_*i*_ = *l*_*i*_ is defined by

(18) \begin{eqnarray*}\widehat{Var} \left( {\hat {\theta }}_{{l}_{i}} \right) = \frac{{ \left( {\hat {\theta }}_{i}-{l}_{i} \right) }^{2}}{{z}_{\alpha /2}^{2}} .\end{eqnarray*}

Substituting back into Eq. (17) as follows

(19) \begin{eqnarray*}L={\hat {\theta }}_{1}+{\hat {\theta }}_{2}-\sqrt{{ \left( {\hat {\theta }}_{1}-{l}_{1} \right) }^{2}+{ \left( {\hat {\theta }}_{2}-{l}_{2} \right) }^{2}}.\end{eqnarray*}

Similarly, the variance estimate for ${\hat {\theta }}_{i}$ at *θ*_*i*_ = *u*_*i*_ is defined by

(20) \begin{eqnarray*}\widehat{Var} \left( {\hat {\theta }}_{{u}_{i}} \right) = \frac{{ \left( {u}_{i}-{\hat {\theta }}_{i} \right) }^{2}}{{z}_{\alpha /2}^{2}} .\end{eqnarray*}

The upper limit *U* is

(21) \begin{eqnarray*}U={\hat {\theta }}_{1}+{\hat {\theta }}_{2}+\sqrt{{ \left( {u}_{1}-{\hat {\theta }}_{1} \right) }^{2}+{ \left( {u}_{2}-{\hat {\theta }}_{2} \right) }^{2}}.\end{eqnarray*}

Therefore, the variance estimate for ${\hat {\theta }}_{i}$ at *θ*_*i*_ = *l*_*i*_ and *θ*_*i*_ = *u*_*i*_ is defined by

(22) \begin{eqnarray*}\widehat{Var} \left( {\hat {\theta }}_{i} \right) = \frac{1}{2} \left( \frac{{ \left( {\hat {\theta }}_{i}-{l}_{i} \right) }^{2}}{{z}_{\alpha /2}^{2}} + \frac{{ \left( {u}_{i}-{\hat {\theta }}_{i} \right) }^{2}}{{z}_{\alpha /2}^{2}} \right) ,\end{eqnarray*}

where *i* = 1, 2.

In this paper, the *k* parameters of interest are *θ*_1_, *θ*_2_, …, *θ*_*k*_. The concepts of Zou & Donner (2008) and Zou, Taleban & Hao (2009) are motivated for constructing the confidence interval for *θ*_1_ + *θ*_2_ + … + *θ*_*k*_ are

(23) \begin{eqnarray*}L={\hat {\theta }}_{1}+{\hat {\theta }}_{2}+\ldots +{\hat {\theta }}_{k}-\sqrt{{ \left( {\hat {\theta }}_{1}-{l}_{1} \right) }^{2}+{ \left( {\hat {\theta }}_{2}-{l}_{2} \right) }^{2}+\ldots +{ \left( {\hat {\theta }}_{k}-{l}_{k} \right) }^{2}}\end{eqnarray*}

and

(24) \begin{eqnarray*}U={\hat {\theta }}_{1}+{\hat {\theta }}_{2}+\ldots +{\hat {\theta }}_{k}+\sqrt{{ \left( {u}_{1}-{\hat {\theta }}_{1} \right) }^{2}+{ \left( {u}_{2}-{\hat {\theta }}_{2} \right) }^{2}+\ldots +{ \left( {u}_{k}-{\hat {\theta }}_{k} \right) }^{2}},\end{eqnarray*}

where (*l*_1_, *u*_1_), (*l*_2_, *u*_2_), …, (*l*_*k*_, *u*_*k*_) contain the parameter values for *θ*_1_, *θ*_2_, …, *θ*_*k*_, respectively.

According to Graybill & Deal (1959), the common coefficient of variation *θ* is weighted average of the coefficient of variation ${\hat {\theta }}_{i}$ based on *k* individual samples. Therefore, the common coefficient of variation is defined by

(25) \begin{eqnarray*}\hat {\theta }=\sum _{i=1}^{k} \frac{{\hat {\theta }}_{i}}{\widehat{Var} \left( {\hat {\theta }}_{i} \right) } \left/ \right. \sum _{i=1}^{k} \frac{1}{\widehat{Var} \left( {\hat {\theta }}_{i} \right) } ,\end{eqnarray*}

where ${\hat {\theta }}_{i}=\sqrt{\exp \left( {S}_{i}^{2} \right) -1}$ and $\widehat{Var} \left( {\hat {\theta }}_{i} \right) $ is defined in Eq. (22).

Applying Krishnamoorthy & Oral (2017), the lower limit and upper limit of the confidence interval for the common coefficient of variation *θ* are defined by

(26) \begin{eqnarray*}{L}_{MOVER}=\hat {\theta }-\sqrt{\sum _{i=1}^{k} \frac{{ \left( {\hat {\theta }}_{i}-{l}_{i} \right) }^{2}}{{ \left( \widehat{Var} \left( {\hat {\theta }}_{{l}_{i}} \right) \right) }^{2}} \left/ \right. \sum _{i=1}^{k} \frac{1}{{ \left( \widehat{Var} \left( {\hat {\theta }}_{{l}_{i}} \right) \right) }^{2}} }\end{eqnarray*}

and

(27) \begin{eqnarray*}{U}_{MOVER}=\hat {\theta }+\sqrt{\sum _{i=1}^{k} \frac{{ \left( {u}_{i}-{\hat {\theta }}_{i} \right) }^{2}}{{ \left( \widehat{Var} \left( {\hat {\theta }}_{{u}_{i}} \right) \right) }^{2}} \left/ \right. \sum _{i=1}^{k} \frac{1}{{ \left( \widehat{Var} \left( {\hat {\theta }}_{{u}_{i}} \right) \right) }^{2}} },\end{eqnarray*}

where $\hat {\theta }$ is defined in Eq. (25).

According to Niwitpong (2013), for *i* = 1, 2, …, *k*, the confidence interval for coefficient of variation of log-normal distribution based on the *i*th sample is given by

(28) \begin{eqnarray*}[{l}_{i},{u}_{i}]= \left[ \right. \sqrt{\exp \nolimits \left( \frac{({n}_{i}-1){S}_{i}^{2}}{{\chi }_{({n}_{i}-1),(1-\alpha /2)}^{2}} \right) -1},\sqrt{\exp \nolimits \left( \frac{({n}_{i}-1){S}_{i}^{2}}{{\chi }_{({n}_{i}-1),(\alpha /2)}^{2}} \right) -1} \left( \right. ,\end{eqnarray*}

where ${\chi }_{({n}_{i}-1),(1-\alpha /2)}^{2}$ and ${\chi }_{({n}_{i}-1),(\alpha /2)}^{2}$ denote the $100 \left( 1-\alpha /2 \right) $-th and $100 \left( \alpha /2 \right) $-th percentiles of the chi-squared distribution with *n*_*i*_ − 1 degrees of freedom.

Therefore, the $100 \left( 1-\alpha \right) \text{%}$ two-sided confidence interval for the common coefficient of variation *θ* based on MOVER approach is

(29) \begin{eqnarray*}C{I}_{MOVER}=[{L}_{MOVER},{U}_{MOVER}],\end{eqnarray*}

where *L*_*MOVER*_ is defined in Eq. (26), *U*_*MOVER*_ is defined in Eq. (27), and *l*_*i*_ and *u*_*i*_ are defined in Eq. (28).

### Computational confidence interval

**Theorem 1:** Let ${Y}_{i}= \left( {Y}_{i1},{Y}_{i2},\ldots ,{Y}_{i{n}_{i}} \right) $ be a log-normal population with parameters *μ*_*i*_ and ${\sigma }_{i}^{2}$, where *i* = 1, 2, …, *k*. For *i* = 1, 2, …, *k* and *j* = 1, 2, …, *n*_*i*_, let ${X}_{ij}=\log \left( {Y}_{ij} \right) $ be the normal distribution with mean *μ*_*i*_ and variance ${\sigma }_{i}^{2}$. The maximum likelihood estimators of *μ*_*i*_ and *θ* given by Eq. (3) under *θ*_1_ = *θ*_2_ = … = *θ*_*k*_ = *θ* are given by

(30) \begin{eqnarray*}{\hat {\mu }}_{i}={\bar {X}}_{i}\end{eqnarray*}

and

(31) \begin{eqnarray*}\sum _{i=1}^{k} \frac{{n}_{i}{\hat {\sigma }}_{i}^{2}}{\log \nolimits \left( {\theta }^{2}+1 \right) } -\sum _{i=1}^{k}{n}_{i}=0.\end{eqnarray*}

**Proof:** The log-likelihood function of normal distribution with parameters *μ*_*i*_ and *θ* is given by

\begin{eqnarray*}\ln \nolimits L=- \frac{1}{2} \sum _{i=1}^{k}{n}_{i}\log \nolimits \left( 2\pi \log \nolimits \left( {\theta }^{2}+1 \right) \right) -\sum _{i=1}^{k}{n}_{i}\log \nolimits \left( {Y}_{ij} \right) -\sum _{i=1}^{k}\sum _{j=1}^{{n}_{i}} \frac{{ \left( \log \nolimits \left( {Y}_{ij} \right) -{\mu }_{i} \right) }^{2}}{2\log \nolimits \left( {\theta }^{2}+1 \right) } . \end{eqnarray*}

Differentiating the ln*L* with respect to *μ*_*i*_ and *θ*, respectively, the maximum likelihood estimators of *μ*_*i*_ and *θ* are given by

${\hat {\mu }}_{i}={\bar {X}}_{i}$

and

\begin{eqnarray*}\sum _{i=1}^{k} \frac{{n}_{i}{\hat {\sigma }}_{i}^{2}}{\log \nolimits \left( {\theta }^{2}+1 \right) } -\sum _{i=1}^{k}{n}_{i}=0. \end{eqnarray*}

Hence, Theorem 1 is proved.

According to Pal, Lim & Ling (2007), the computational approach uses the maximum likelihood estimates (MLEs). The common coefficient of variation based on maximum likelihood estimator is defined by

(32) \begin{eqnarray*}{\hat {\theta }}_{ML}=\sum _{i=1}^{k} \frac{{\hat {\theta }}_{i}}{\widehat{Var}({\hat {\theta }}_{i})} \left/ \right. \sum _{i=1}^{k} \frac{1}{\widehat{Var}({\hat {\theta }}_{i})} ,\end{eqnarray*}

where ${\hat {\theta }}_{i}=\sqrt{\exp \left( {S}_{i}^{2} \right) -1}$ and $\widehat{Var} \left( {\hat {\theta }}_{i} \right) $ is defined in Eq. (4) with *σ*_*i*_ replaced by *s*_*i*_.

The computational approach is to obtain the restricted maximum likelihood estimates (RMLEs) of parameters. The maximum likelihood estimators of *μ*_*i*_ and *θ* under *θ*_1_ = *θ*_2_ = … = *θ*_*k*_ = *θ* provide the RMLEs of these parameters.

Then the RMLE of *μ*_*i*_ is defined by ${\hat {\mu }}_{i(RML)}={\bar {X}}_{i}$. The RMLE of *θ* obtained iteratively from Eq. (31) by using bisection method. The *θ* converge to the RMLE denoted as ${\hat {\theta }}_{RML}$.

Data replication from $f(x{|}{\hat {\mu }}_{i(RML)},{\hat {\theta }}_{RML})$ is used to construct the confidence interval based on computational approach. Let artificial sample ${X}_{i(RML)}= \left( {X}_{i1(RML)},{X}_{i2(RML)},\ldots ,{X}_{i{n}_{i}(RML)} \right) $ be the normal distribution with mean ${\hat {\mu }}_{i(RML)}$ and variance ${\hat {\sigma }}_{i(RML)}^{2}$. Let ${\bar {X}}_{i(RML)}$ and ${S}_{i(RML)}^{2}$ be the mean and variance of the log-transformed sample from a log-normal distribution for the *i*th artificial sample and let ${\bar {x}}_{i(RML)}$ and ${s}_{i(RML)}^{2}$ be observed sample mean and observed sample variance, respectively.

Therefore, the common coefficient of variation based on *k* individual samples is defined by

(33) \begin{eqnarray*}{\hat {\theta }}_{RML}=\sum _{i=1}^{k} \frac{{\hat {\theta }}_{i(RML)}}{\widehat{Var}({\hat {\theta }}_{i(RML)})} \left/ \right. \sum _{i=1}^{k} \frac{1}{\widehat{Var}({\hat {\theta }}_{i(RML)})} ,\end{eqnarray*}

where ${\hat {\theta }}_{i(RML)}=\sqrt{\exp \left( {S}_{i(RML)}^{2} \right) -1}$.

Therefore, the $100 \left( 1-\alpha \right) \text{%}$ two-sided confidence interval for the common coefficient of variation *θ* based on computational approach is

(34) \begin{eqnarray*}C{I}_{CA}=[{L}_{CA},{U}_{CA}]=[{\hat {\theta }}_{RML} \left( \alpha /2 \right) ,{\hat {\theta }}_{RML} \left( 1-\alpha /2 \right) ],\end{eqnarray*}

where ${\hat {\theta }}_{RML} \left( \alpha /2 \right) $ and ${\hat {\theta }}_{RML} \left( 1-\alpha /2 \right) $ denote the $ \left( \alpha /2 \right) $-th and $ \left( 1-\alpha /2 \right) $-th percentiles of ${\hat {\theta }}_{RML}$, respectively.

The following algorithm is used to construct the computational confidence interval:


 
 
   Algorithm 2 
For a given ¯ xi, s2i, and θ, where i = 1,2,...,k 
Compute ˆ μi(RML)  and ˆ θRML  from Eqs.  (30)--31 
For g =  1 to m 
Generate xij(RML)  from N ( 
  ˆ μi(RML),∘ 
  __________log ( 
   ˆ θ2 
RML + 1) 
            ) 
Compute ¯ xi(RML)  and s2i(RML) 
Compute ˆ θRML  from Eq.  (33) 
End g loop 
Compute ˆ θRML (α∕2)  and ˆ θRML (1 − α∕2)  from Eq.  (34)    


### Bayesian confidence interval

The FGCI approach, MOVER approach, and computational approach are the classical approach. The classical approach and the Bayesian approach are fundamentally different. In the classical approach, the parameter of interest *θ* is unknown, but it is fixed. In the Bayesian approach, the parameter is considered to be a quantity. The variation of the quantity is described by the prior distribution. Bayes (1763) introduced that Bayesian approach uses Bayes’ theorem to update probabilities. Bayes’ theorem describes the conditional probability of an event based on data. The data is prior information or beliefs about the event. The posterior distribution is combination of the likelihood function and the prior distribution. The Bayesian confidence interval is constructed based on the posterior distribution. The posterior distribution is a conditional distribution which is based on the observed values of the sample. The posterior distribution is used to make statements about the parameter. The parameter is considered a random quantity. The conditional posterior distribution for *μ*_*i*_ given ${\sigma }_{i}^{2}$ and *x*_*i*_ is the normal distribution with mean ${\hat {\mu }}_{i}$ and variance ${\sigma }_{i}^{2}/{n}_{i}$. The distribution is defined by

(35) \begin{eqnarray*}{\mu }_{i}{|}{\sigma }_{i}^{2},{x}_{i}\sim N({\hat {\mu }}_{i},{\sigma }_{i}^{2}/{n}_{i}).\end{eqnarray*}

The posterior distribution for ${\sigma }_{i}^{2}$ is inverse gamma distribution. It is defined by

(36) \begin{eqnarray*}{\sigma }_{i}^{2}{|}{x}_{i}\sim IG(({n}_{i}-1)/2,({n}_{i}-1){s}_{i}^{2}/2).\end{eqnarray*}

The posterior distribution of coefficient of variation of log-normal distribution is

(37) \begin{eqnarray*}{\hat {\theta }}_{i}=\sqrt{\exp \nolimits \left( {\sigma }_{i}^{2} \right) -1},\end{eqnarray*}

where ${\sigma }_{i}^{2}$ is defined in Eq. (36).

The variance of ${\hat {\theta }}_{i}$ is

(38) \begin{eqnarray*}Var({\hat {\theta }}_{i})\approx \frac{{\sigma }_{i}^{4}(\exp \nolimits (2{\sigma }_{i}^{2}))}{2({n}_{i}-1)(\exp \nolimits ({\sigma }_{i}^{2})-1)} ,\end{eqnarray*}

where ${\sigma }_{i}^{2}$ is defined in Eq. (36).

The common coefficient of variation of log-normal distribution based on *k* individual samples which the parameter of interest defined by

(39) \begin{eqnarray*}{\hat {\theta }}_{BS}=\sum _{i=1}^{k} \frac{{\hat {\theta }}_{i}}{Var({\hat {\theta }}_{i})} \left/ \right. \sum _{i=1}^{k} \frac{1}{Var({\hat {\theta }}_{i})} ,\end{eqnarray*}

where $({\hat {\theta }}_{i})$ is defined in Eq. (37) and $Var({\hat {\theta }}_{i})$ is defined in Eq. (38).

Gelman et al. (2013) introduced the highest posterior density interval to construct the Bayesian confidence interval. Therefore, the $100 \left( 1-\alpha \right) \text{%}$ two-sided confidence interval for the common coefficient of variation *θ* based on Bayesian approach is

(40) \begin{eqnarray*}C{I}_{BS}=[{L}_{BS},{U}_{BS}],\end{eqnarray*}

where *L*_*BS*_ and *U*_*BS*_ are the lower limit and the upper limit of the shortest $100 \left( 1-\alpha \right) \text{%}$ highest posterior density interval of ${\hat {\theta }}_{BS}$, respectively.

The following algorithm is used to construct the Bayesian confidence interval:


 
 
   Algorithm 3 
For a given ¯ xi  and s2i, where i = 1,2,...,k 
For g =  1 to m 
Generate μi|σ2i,xi ∼ N(ˆμi,σ2i∕ni) 
Generate σ2i|xi ∼ IG((ni − 1)∕2,(ni − 1)s2i∕2) 
Compute θi  from Eq.  (37) 
Compute V ar(ˆθi)  from Eq.  (38) 
Compute θBS  from Eq.  (39) 
End g loop 
Compute LBS  and UBS    


## Results

A simulation study was performed to evaluate the coverage probabilities and average lengths of the FGCI (*CI*_*FGCI*_), MOVER (*CI*_*MOVER*_), computational (*CI*_*CA*_), and Bayesian confidence intervals (*CI*_*BS*_). The confidence intervals were compared by measuring their coverage probabilities and average lengths and, in each case, the one with a coverage probability closest to the nominal confidence level (1 − *α*) and with the shortest average length was chosen as the most appropriate.

In this simulation study, the nominal confidence level was chosen as 0.95. The sample cases used were *k* = 3 and *k* = 6 with sample sizes *n*_1_, *n*_2_, …, *n*_*k*_, as in Tables 1 and 2. Following Tian & Wu (2007) and Ng (2014), the coefficient of variation of log-normal distribution, which equals $\sqrt{\exp \left( {\sigma }^{2} \right) -1}$, is chosen in the range from 0.05 - 2.00. Since the coefficient of variation of a log-normal distribution is independent of µ, the population means of the normal data within each sample were given the same value *μ*_1_ = *μ*_2_ = … = *μ*_*k*_ = *μ* = 1 to simplify matters, and the population standard deviations *σ*_1_, *σ*_2_, …, *σ*_*k*_ are as in Tables 1 and 2. For each parameter setting, 5,000 random samples were generated by applying Algorithm 4, and thus 1000*R*_*θ*_, 1000${\hat {\theta }}_{RML}$, and 1000*θ*_*BS*_ were simulated by applying Algorithm 1, 2, and 3, respectively, for each of the random samples.

**Table 1:** The coverage probabilities and average lengths of 95% two-sided confidence intervals for the common coefficient of variation of several log-normal populations: three sample cases.

**(*n*_1_, *n*_2_, *n*_3_)**	**(*σ*_1_, *σ*_2_, *σ*_3_)**	**Coverage probability (Average length)**			
		***CI*_*FGCI*_**	***CI*_*MOVER*_**	***CI*_*CA*_**	***CI*_*BS*_**
(30,30,30)	(0.05,0.10,0.15)	0.9514	0.9238	0.9188	0.9400
		(0.0317)	(0.0306)	(0.0305)	(0.0309)
	(0.50,1.00,1.00)	0.9496	0.9150	0.9246 )	0.9394
		(0.3922)	(0.3672)	(0.3628	(0.3781)
(50,50,50)	(0.05,0.10,0.15)	0.9492	0.9234	0.9244	0.9408
		(0.0246)	(0.0230)	(0.0240)	(0.0241)
	(0.50,1.00,1.00)	0.9504	0.9054	0.9372	0.9442
		(0.3060)	(0.2707)	(0.2909)	(0.2976)
(30,50,100)	(0.05,0.10,0.15)	0.9530	0.9160	0.9200	0.9426
		(0.0331)	(0.0312)	(0.0329)	(0.0325)
	(0.50,1.00,1.00)	0.9498	0.8808	0.9186	0.9360
		(0.4259)	(0.3831)	(0.4055)	(0.4147)
(50,100,200)	(0.05,0.10,0.15)	0.9528	0.9222	0.9358	0.9430
		(0.0251)	(0.0234)	(0.0252)	(0.0247)
	(0.50,1.00,1.00)	0.9492	0.8722	0.9314	0.9410
		(0.3357)	(0.2875)	(0.3272)	(0.3291)
(100,100,100)	(0.05,0.10,0.15)	0.9502	0.9214	0.9388	0.9442
		(0.0174)	(0.0159)	(0.0172)	(0.0171)
	(0.50,1.00,1.00)	0.9478	0.8952	0.9398	0.9394
		(0.2176)	(0.1852)	(0.2117)	(0.2129)
(200,200,200)	(0.05,0.10,0.15)	0.9446	0.9178	0.9396	0.9404
		(0.0123)	(0.0111)	(0.0122)	(0.0121)
	(0.50,1.00,1.00)	0.9488	0.8944	0.9456	0.9434
		(0.1542)	(0.1288)	(0.1520)	(0.1513)
(500,500,500)	(0.05,0.10,0.15)	0.9482	0.9200	0.9444	0.9420
		(0.0078)	(0.0070)	(0.0078)	(0.0077)
	(0.50,1.00,1.00)	0.9480	0.8940	0.9470	0.9430
		(0.0976)	(0.0807)	(0.0971)	(0.0961)
(1000,1000,1000)	(0.05,0.10,0.15)	0.9428	0.9162	0.9446	0.9390
		(0.0055)	(0.0049)	(0.0055)	(0.0054)
	(0.50,1.00,1.00)	0.9574	0.9020	0.9552	0.9524
		(0.0691)	(0.0569)	(0.0689)	(0.0681)

**Table 2:** The coverage probabilities and average lengths of 95% two-sided confidence intervals for the common coefficient of variation of several log-normal populations: six sample cases.

**(*n*_1_, *n*_2_, *n*_3_, *n*_4_, *n*_5_, *n*_6_)**	**(*σ*_1_, *σ*_2_, *σ*_3_, *σ*_4_, *σ*_5_, *σ*_6_)**	**Coverage probability (Average length)**			
		***CI*_*FGCI*_**	***CI*_*MOVER*_**	***CI*_*CA*_**	***CI*_*BS*_**
(30,30,30,30,30,30)	(0.05,0.05,0.10,0.10,0.15,0.15)	0.9432	0.9818	0.8812	0.9328
		(0.0226)	(0.0298)	(0.0227)	(0.0222)
	(0.50,0.50,0.50,1.00,1.00,1.00)	0.9218	0.9838	0.8418	0.8982
		(0.2099)	(0.3278)	(0.2108)	(0.2054)
(30,50,100,30,50,100)	(0.05,0.05,0.10,0.10,0.15,0.15)	0.9400	0.9800	0.8928	0.9314
		(0.0199)	(0.0247)	(0.0202)	(0.0196)
	(0.50,0.50,0.50,1.00,1.00,1.00)	0.9350	0.9830	0.8992	0.9224
		(0.1490)	(0.2033)	(0.1505)	(0.1461)
(50,50,50,50,50,50)	(0.05,0.05,0.10,0.10,0.15,0.15)	0.9392	0.9834	0.9060	0.9342
		(0.0175)	(0.0227)	(0.0175)	(0.0172)
	(0.50,0.50,0.50,1.00,1.00,1.00)	0.9290	0.9890	0.8812	0.9182
		(0.1628)	(0.2503)	(0.1638)	(0.1598)
(30,30,50,50,100,100)	(0.05,0.05,0.10,0.10,0.15,0.15)	0.9354	0.9808	0.8636	0.9218
		(0.0239)	(0.0305)	(0.0245)	(0.0235)
	(0.50,0.50,0.50,1.00,1.00,1.00)	0.9218	0.9800	0.8596	0.9048
		(0.2128)	(0.2931)	(0.2148)	(0.2086)
(50,50,100,100,200,200)	(0.05,0.05,0.10,0.10,0.15,0.15)	0.9432	0.9860	0.9028	0.9360
		(0.0180)	(0.0231)	(0.0184)	(0.0178)
	(0.50,0.50,0.50,1.00,1.00,1.00)	0.9354	0.9822	0.9008	0.9218
		(0.1598)	(0.2103)	(0.1611)	(0.1571)
(30,30,50,100,100,200)	(0.05,0.05,0.10,0.10,0.15,0.15)	0.9398	0.9800	0.8678	0.9312
		(0.0226)	(0.0283)	(0.0236)	(0.0223)
	(0.50,0.50,0.50,1.00,1.00,1.00)	0.9284	0.9788	0.8618	0.9100
		(0.2257)	(0.2980)	(0.2291)	(0.2216)
(100,100,100,100,100,100)	(0.05,0.05,0.10,0.10,0.15,0.15)	0.9480	0.9830	0.9282	0.9406
		(0.0123)	(0.0158)	(0.0124)	(0.0122)
	(0.50,0.50,0.50,1.00,1.00,1.00)	0.9362	0.9952	0.9166	0.9256
		(0.1149)	(0.1747)	(0.1154)	(0.1131)
(200,200,200,200,200,200)	(0.05,0.05,0.10,0.10,0.15,0.15)	0.9512	0.9872	0.9410	0.9428
		(0.0087)	(0.0111)	(0.0087)	(0.0086)
	(0.50,0.50,0.50,1.00,1.00,1.00)	0.9418	0.9968	0.9322	0.9364
		(0.0813)	(0.1231)	(0.0816)	(0.0801)
(500,500,500,500,500,500)	(0.05,0.05,0.10,0.10,0.15,0.15)	0.9510	0.9876	0.9440	0.9464
		(0.0055)	(0.0070)	(0.0055)	(0.0054)
	(0.50,0.50,0.50,1.00,1.00,1.00)	0.9520	0.9960	0.9494	0.9494
		(0.0514)	(0.0776)	(0.0514)	(0.0506)
(1000,1000,1000,1000,1000,1000)	(0.05,0.05,0.10,0.10,0.15,0.15)	0.9520	0.9890	0.9496	0.9490
		(0.0039)	(0.0049)	(0.0039)	(0.0038)
	(0.50,0.50,0.50,1.00,1.00,1.00)	0.9520	0.9974	0.9488	0.9470
		(0.0363)	(0.0548)	(0.0363)	(0.0358)

The following algorithm is used to estimate the coverage probability and average length:


 
 
     Algorithm 4 
For a given (n1,n2,...,nk), (μ1,μ2,...,μk), (σ1,σ2,...,σk)  and θ 
For h =  1 to M 
Generate xij  from N(μi,σ2i), where i = 1,2,...,k and j = 1,2,...,ni 
Calculate ¯ xi  and s2i 
Construct [LFGCI(h),UFGCI(h)] 
Construct [LMOV ER(h),UMOV ER(h)] 
Construct [LCA(h),UCA(h)] 
Construct [LBS(h),UBS(h)] 
Record whether or not all the values of θ fall in their correspond- 
ing confidence intervals 
Compute U(h) − L(h) 
End h loop 
Compute the coverage probability and the average length for each con- 
fidence interval    


Tables 1 and 2 report the coverage probabilities and average lengths for *k* = 3 and *k* = 6, respectively. From Table 1, the simulation results indicate that for all sample sizes, the FGCI approach provided the best coverage probabilities whereas the MOVER confidence interval attained coverage probabilities under the nominal confidence level of 0.95. Furthermore, the coverage probabilities of the MOVER confidence interval decreased when the sample sizes increased. The computational confidence interval achieved coverage probabilities under the nominal confidence level of 0.95, which became closer to it as the sample sizes increased. The coverage probabilities of the Bayesian confidence interval performed well when the population standard deviations were (0.05,0.10,0.15) whereas they were less than the nominal confidence level of 0.95 when the population standard deviations were (0.50,1.00,1.00).

From Table 2, it can be seen that the coverage probabilities of the FGCI and the computational confidence interval were less than the nominal confidence level of 0.95 when the sample sizes were small. For large sample sizes, the coverage probabilities of the FGCI and the computational confidence interval were close to the nominal confidence level of 0.95, with those of the FGCIs being closer. The coverage probabilities of the MOVER confidence interval were greater than the nominal confidence level of 0.95 for small sample sizes and became close to 1.00 when the sample sizes increased, thereby showing conservative behavior when the sample case (*k*) was large and the sample sizes were large. Therefore, the MOVER approach can be considered as an alternative to estimate the confidence interval for the common coefficient of variation of log-normal distributions when the sample case (*k* = 6) is large and the sample sizes are small. The coverage probabilities of the Bayesian confidence interval were less than the nominal confidence level of 0.95 when the sample sizes were small and were close to the nominal confidence level of 0.95 when the sample sizes were large. The average lengths of the Bayesian confidence interval were shorter than those of the others.

As the sample case (*k*) increased, the coverage probabilities of the FGCI and the computational confidence interval tended to decrease because the common coefficient of variation *θ* is based on the variance of the coefficient of variation ${\hat {\theta }}_{i}$ for the *k* individual samples. Herein, we present only the results of *μ* = 1 because they tended toward the same direction regardless of the value of µ. In all cases, the coverage probabilities were affected by large *σ* values because the coefficient of variation of log-normal distributions $\theta =\sqrt{\exp \left( {\sigma }^{2} \right) -1}$ depends on parameter *σ* only.

###  An empirical application

The rainfall has been the seasonal problem in Thailand. Rainy season in Thailand is between May and October. The daily rainfall appears on 17 June 2020. Real data example of rainfall data is used to illustrate the FGCI, MOVER, computational, and Bayesian approaches. All data were reported by the Thai Meteorological Department (https://www.tmd.go.th/climate/climate.php).

**Figure 1:**
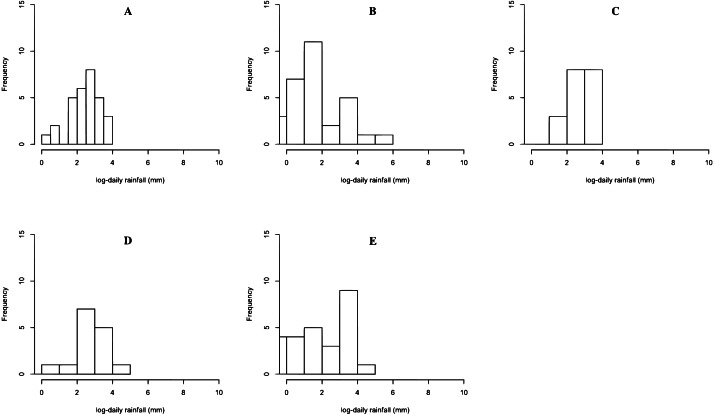
Histogram of rainfall data in (A) Northern (B) Northeastern (C) Central (D) Eastern (E) Southern regions.

**Figure 2:**
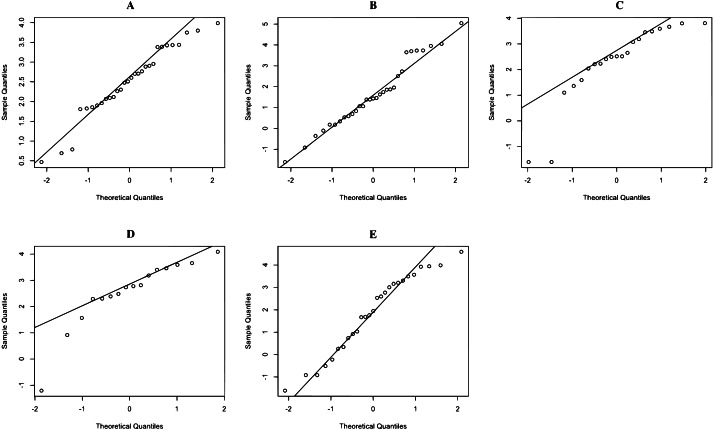
QQ-plot of log- rainfall data in (A) Northern (B) Northeastern (C) Central (D) Eastern (E) Southern regions

The rainfall data on 17 June 2020 in Northern, Northeastern, Central, Eastern, and Southern regions are reported in Dataset 1, and histogram and normal QQ-plots are presented in Figs. 1 and 2, respectively. The data sets consist of 30 measurements in Northern region, 31 measurements in Northeastern region, 21 measurements in Central region, 16 measurements in Eastern region, and 27 measurements in Southern region. The statistics is summarized in Table 3. The Shapiro–Wilk normality test is used to check the assumption that the log-data is normal distribution. The Shapiro–Wilk normality test with *p*-values 0.2176, 0.4981, 0.0009, 0.0128, and 0.2127 for Northern, Northeastern, Central, Eastern, and Southern regions, respectively. From *p*-values, the results show that the rainfall of the three regions follow log-normal distributions such as Northern, Northeastern, and Southern regions. The data of Northern, Northeastern, and Southern regions were used to construct the confidence interval for the common coefficient of variation based on the four approaches. The true common coefficient of variation was 1.2084. The point estimators of the common coefficient of variation based on the FGCI, MOVER, CA, and Bayesian approaches were 1.2776, 1.1486, 1.2273, and 1.2669. The FGCI, MOVER, computational, and Bayesian confidence intervals were [0.8380, 2.0301] with an interval length of 1.1921, [0.8460, 1.8481] with an interval length of 1.0021, [0.7745, 1.8663] with an interval length of 1.0918, and [0.7991, 1.7704] with an interval length of 0.9713, respectively. Hence, the length of the Bayesian confidence interval was shorter than those of the others, and so was more accurate.

## Discussion

As a limitation of this study, the coefficient of variation of log-normal distribution can be computed directly from log-normal data and the data transformed using the log function. The coefficients of variation based directly on log-normal data make it easy to construct the confidence interval for the common coefficient of variation of log-normal distributions because the coefficient of variation of a log-normal distribution is based on *σ*^2^ only. However, for a greater number of samples, i.e., *n* = 500 and a large *σ*^2^, i.e., *σ*^2^ = 10, the coverage probabilities of all of the proposed confidence intervals cannot be computed (the simulation results are not reported here). The coefficients of variation based on transformed data using the log function make it more difficult to estimate the confidence interval for the common coefficient of variation of log-normal distributions because the coefficient of variation of a normal distribution is based on both µ and *σ*^2^.

The MOVER approach makes it possible to compute the confidence interval online because this approach requires a simple formula to construct the confidence interval. Since the FGCI, computational, and Bayesian approaches are based on simulation techniques, it is not possible to compute the confidence intervals for them online.

As a final note, the results of the computational approach did not perform well for the confidence interval estimation for the common coefficient of variation of log-normal distributions. However, Thangjai, Niwitpong & Niwitpong (2020b) reported that it performs well for constructing the confidence interval for the common coefficient of variation of normal distributions when the sample case is large.

**Table 3:** Sample statistics of Northern, Northeastern, Central, Eastern, and Southern regions (mm).

**Statistics**	**Northern region**	**Northeastern region**	**Central region**	**Eastern region**	**Southern region**
*n*	30	31	21	16	27
$\bar {y}$	17.1200	16.8677	17.9333	19.9688	18.3852
*s*_*Y*_	13.5250	30.9424	14.7627	15.8678	23.2974
$\bar {x}$	2.5082	1.6238	2.2851	2.5292	1.8608
*s*_*X*_	0.8945	1.6007	1.5163	1.2827	1.7549
$\hat {\theta }$	1.1072	3.4592	2.9942	2.0450	4.5550

## Conclusions

The results in Tables 1 and 2 indicate that the FGCI approach provided much better confidence interval estimates than the other approaches in terms of coverage probability for almost all sample cases (*k*) and sample sizes (*n*), except that the MOVER approach provided the best confidence interval estimates when the sample case was equal to six populations (*k* = 6) and the sample sizes were small (*n*_*i*_ < 50). Moreover, the FGCI approach was the best for constructing the confidence interval for all sample sizes (*n*) when the sample case is small (*k* = 3), for which the coverage probability of the FGCI approach was stable around the nominal confidence level of 0.95. For large sample cases (*k* = 6), the FGCI approach performed well for confidence interval estimation when the sample sizes were large. Furthermore, the FGPQ for the FGCI approach is not dependent on the population variances (*σ*_*i*_). The results are similar to those of Hannig et al. (2006b), Chang & Huang (2009), Kharrati-Kopaei, Malekzadeh & Sadooghi-Alvandi (2013), and Thangjai, Niwitpong & Niwitpong (2016). Note that the GCIs based on Ng (2014) had coverage probabilities close to 1.00 when the sample size was small and close to the nominal confidence level of 0.95 when the sample size was large. Therefore, the GCIs based on Ng (2014) are rather conservative for small sample sizes.

##  Supplemental Information

10.7717/peerj.10004/supp-1 Supplemental Information 1 R code for computing an application data

10.7717/peerj.10004/supp-2 Supplemental Information 2 R code for running coverage probability and average length width of all confidence intervals

10.7717/peerj.10004/supp-3 Supplemental Information 3 Rainfall data of Northern, Northeastern, Central, Eastern, and Southern regions (mm)

## Additional Information and Declarations

### Data Availability

The following information was supplied regarding data availability: Raw data and code are available in the Supplemental Files.
