# Supplementary material for: Confidence intervals for the common coefficient of variation of rainfall in Thailand
Source: PeerJ. 2020 Sep 21;8:e10004. doi: 10.7717/peerj.10004 (PMC7513754; doi:10.7717/peerj.10004)
Supplement: Supplemental Information 3 [file peerj-08-10004-s003.docx]

**Dataset S1.** Rainfall data of Northern, Northeastern, Central, Eastern, and Southern regions (mm)

| **Northern region** | | **Northeastern region** | | **Central**  **region** | | **Eastern**  **region** | | **Southern region** | |
| --- | --- | --- | --- | --- | --- | --- | --- | --- | --- |
| 2.2 | 19.2 | 7.1 | 4.0 | 0.2 | 4.9 | 36.4 | 31.8 | 0.8 | 2.1 |
| 42.4 | 53.8 | 6.5 | 6.4 | 9.1 | 24.2 | 16.7 | 4.8 | 23.6 | 0.6 |
| 7.1 | 2.0 | 1.4 | 0.4 | 32.8 | 45.2 | 10.9 | 59.7 | 0.4 | 1.3 |
| 31.1 | 6.1 | 2.9 | 4.2 | 21.7 | 11.1 | 12.0 | 38.8 | 5.3 | 15.9 |
| 12.3 | 14.9 | 4.0 | 155.4 | 9.3 | 12.1 | 0.3 | 30.0 | 50.5 | 2.8 |
| 1.6 | 17.9 | 0.7 | 1.8 | 3.0 | 44.8 | 16.1 | 2.5 | 0.2 | 0.4 |
| 30.7 | 29.4 | 2.3 | 0.2 | 12.4 | 0.2 | 15.4 | 9.9 | 7.0 | 35.4 |
| 18.3 | 7.9 | 1.2 | 4.3 | 12.4 | 31.7 | 10.0 | 24.2 | 13.4 | 53.9 |
| 15.9 | 29.6 | 57.7 | 2.9 | 36.4 | 39.3 |  |  | 24.7 | 2.5 |
| 44.6 | 11.9 | 0.9 | 1.7 | 14.2 | 7.7 |  |  | 27.2 | 32.8 |
| 8.3 | 6.2 | 52.3 | 41.8 | 3.9 |  |  |  | 51.6 | 5.4 |
| 6.7 | 13.5 | 5.1 | 5.7 |  |  |  |  | 20.2 | 98.6 |
| 15.0 | 10.0 | 40.5 | 42.1 |  |  |  |  | 12.6 | 5.8 |
| 9.6 | 30.9 | 38.5 | 2.0 |  |  |  |  | 1.4 |  |
| 6.4 | 8.1 | 12.3 | 1.2 |  |  |  |  |  |  |
|  |  | 15.4 |  |  |  |  |  |  |  |

**Source:** Thai Meteorological Department (https://www.tmd.go.th/climate/climate.php)
